# Aposentadoria e depressão: perspectiva para clínica ampliada

**DOI:** 10.15446/rsap.V25n2.81109

**Published:** 2023-03-01

**Authors:** Marcela Barretto Costa, Randson Souza Rosa, Tarcísio Pereira Guedes, Rita Narriman Silva de Oliveira Boery, Isleide Santana Cardoso Santos, Jean Carlos Zambrano Contreras, Andréa dos Santos Souza, Jefferson Meira Pires, Lélia Lessa Teixeira Pinto, Tatiane Tavares Reis, Tâmara Souza Santos, José de Bessa

**Affiliations:** 1 MB: Psicól. Graduada em Psicologia. Faculdade de Tecnologia e Ciências (FTC). Jequié/BA, Brasil. marcelapsicoftc@gmail.com Faculdade de Tecnologia e Ciência Faculdade de Tecnologia e Ciência Jequié BA Brasil; 2 RS: Enf. M.Sc. Ciências da Saúde. Programa de Pós-Graduação em Enfermagem e Saúde (PPGES) da Universidade Estadual do Sudoeste da Bahia (UESB), Jequié/BA, Brasil. enfrandson@gmail.com Universidade Estadual do Sudoeste da Bahia Ciências da Saúde Programa de Pós-Graduação em Enfermagem e Saúde Universidade Estadual do Sudoeste da Bahia Jequié BA Brasil; 3 TP: Psicól. Pós-Graduado em Psicanálise Clínica. Faculdade Alvorada Paulista. Docente da Faculdade de Tecnologia e Ciências (FTC). Universidade Estadual de Feira de Santana. tguedes.jeq@ftc.edu.br Faculdade de Tecnologia e Ciências Faculdade de Tecnologia e Ciências tguedes.jeq@ftc.edu.br; 4 RB: Enf. Pós-Ph.D Bioética. Doutora em Enfermagem. Universidade Federal de São Paulo (UNIFESP). Docente do Programa de Pós-Graduação em Enfermagem e Saúde (PPGES) da Universidade Estadual do Sudoeste da Bahia (UESB), Jequié/BA, Brasil. rboery@gmail.com Universidade Estadual do Sudoeste da Bahia Programa de Pós-Graduação em Enfermagem e Saúde Universidade Estadual do Sudoeste da Bahia Jequié BA Brasil; 5 IS: Enf. Ph.D. Ciências da Saúde. Docente, Universidade Estadual do Sudoeste da Bahia (UESB). Jequié/BA, Brasil. isantana@uesb.edu.br Universidade Estadual do Sudoeste da Bahia Universidade Estadual do Sudoeste da Bahia Jequié BA Brasil isantana@uesb.edu.br; 6 JZ: Ed. Fís. Ph.D. Ciências da Atividade Física e o Esporte. Investigador, Universidade Estadual de Feira de Santana (UEFS). Feira de Santan/BA, Brasil. zambrano.jeancarlos@gmail.com Universidade Estadual de Feira de Santana Ciências da Atividade Física e o Esporte Universidade Estadual de Feira de Santana Feira de Santan BA Brasil; 7 AS: Enf. Ph.D. Enfermagem. Docente, Universidade Estadual de Santa Cruz (UESC). Ilheus/BA, Brasil. assouza@uesc.br Universidade Estadual de Santa Cruz Universidade Estadual de Santa Cruz Ilheus BA Brasil assouza@uesc.br; 8 JM: MD. M.Sc. Medicina. Docente, Universidade Estadual do Sudoeste da Bahia (UESB). Jequié, Bahia. uedjat@hotmail.com Universidade Estadual do Sudoeste da Bahia Universidade Estadual do Sudoeste da Bahia Bahia; 9 LP: Ed. Fís. Ph.D. Ciência da Saúde. Programa de Pós-Graduação em Enfermagem e Saúde (PPGES) da Universidade Estadual do Sudoeste da Bahia (UESB). Jequié/BA, Brasil. lelia_lessa@hotmail.com Universidade Estadual do Sudoeste da Bahia Programa de Pós-Graduação em Enfermagem e Saúde Universidade Estadual do Sudoeste da Bahia Jequié BA Brasil; 10 TT: Psicól. M.Sc. Ciências da Saúde. Programa de Pós-Graduação em Enfermagem e Saúde (PPGES) da Universidade Estadual do Sudoeste da Bahia (UESB), Jequié/BA, Brasil. ttreisl6@hotmail.com Universidade Estadual do Sudoeste da Bahia Programa de Pós-Graduação em Enfermagem e Saúde Universidade Estadual do Sudoeste da Bahia Jequié BA Brasil; 11 TS: Enf. M.Sc. Ciências da Saúde. Investigadora, Universidade Estadual do Sudoeste da Bahia (UESB). Jequié/BA, Brasil. tamarasouza65@hotmail.com Universidade Estadual do Sudoeste da Bahia Universidade Estadual do Sudoeste da Bahia Jequié BA Brasil; 12 JB: MD. Ph.D. Ciências. Docente, Universidade Estadual de Feira de Santana (UEFS). Feira de Santana/BA, Brasil. bessa@uefs.br Universidade Estadual de Feira de Santana Universidade Estadual de Feira de Santana Feira de Santana BA Brasil bessa@uefs.br

**Keywords:** Envelhecimento, aposentadoria, depressão, pesquisa interdisciplinar *(fonte: DeCS, BIREME)*, Aging, retirement, depression, interdisciplinary research *(source: MeSH, NLM)*, Envejecimiento, jubilación, depresión, investigación interdisciplinaria *(fuente: DeCS, BIREME)*

## Abstract

**Objetivo:**

O objetivo do estudo foi discutir a relação entre aposentadoria e depressão em idosos promovendo uma reflexão teórica na perspectiva da clínica ampliada diante dessa transição.

**Métodos:**

Reflexão teórica, baseada em artigos científicos que discutem sobre aposentadoria e depressão, enfatizando as percepções e significados do trabalho na velhice, a transição para a aposentadoria, as relações entre aposentadoria e depressão na terceira idade e as contribuições da clínica ampliada na vida do idoso pré e pós-aposentadoria.

**Resultados:**

O presente trabalho foi construído por evidências cientificas, os quais foram selecionados obedecendo os critérios de inclusão e exclusão entre os anos 2012 a 2017, resultando em 15 artigos para discussão dessa reflexão.

**Conclusão:**

Dessa forma, a literatura aponta a necessidade do preparo continuado ao trabalhador e não somente nas vésperas da aposentadoria. Portanto, estas ações de intervenção serão essenciais para que o sujeito idoso possa desfrutar de uma aposentadoria bem sucedida e sem grandes impactos na sua saúde.

## Abstract (en)

**Objective:**

The objective of the study was to discuss the relationship between retirement and depression in the elderly and promoting theoretical reflection from the perspective of the expanded clinic in the face of this transition.

**Method:**

Theoretical reflection, based on scientific articles that discuss retirement and depression, emphasizing the perceptions and meanings of work in old age, the transition to retirement, the relationships between retirement and depression in old age and the contributions of the expanded clinic in the life of the elderly pre and post-retirement.

**Results:**

This work was built on scientific evidence, which were selected following the inclusion and exclusion criteria between 2012 and 2017, resulting in 15 articles to dis-cuss this reflection.

**Conclusions:**

In this way, the literature points out the necessity of continuous preparation for the worker and not only on the eve of retirement. Therefore, these intervention actions will be essential so that the elderly subject can enjoy a successful retirement without major impacts on their health.

## RESUMEN

**Objetivo:**

El objetivo del estudio fue discutir la relación entre jubilación y depresión en ancianos y promover la reflexión teórica desde la perspectiva de la clínica ampliada ante esta transición.

**Método:**

Reflexión teórica, basada en artículos científicos que discuten jubilación y depresión, enfatizando las percepciones y significados del trabajo en la vejez, la transición a la jubilación, las relaciones entre jubilación y depresión en la vejez y los aportes de la clínica ampliada en la vida del Ancianos pre y post-jubilación.

**Resultados:**

Este trabajo se basó en evidencia científica, que fueron seleccionados siguiendo los criterios de inclusión y exclusión entre 2012 y 2017, resultando 15 artículos para discutir esta reflexión.

**Conclusión:**

La literatura apunta a la necesidad de una preparación continua para el trabajador y no solo en la víspera de la jubilación. Por lo tanto, estas acciones de intervención serán esenciales para que las personas de edad avanzada puedan disfrutar de una jubilación exitosa y sin mayores impactos en su salud.

Oaumento da população idosa no Brasil tem crescido de forma significante. Em 2000, a população de idosos com 80 anos ou mais era de 1,6 milhões, espera-se que em 2050 esse número aumente para aproximadamente 13,8 milhões de idosos 1. Esse aumento tem relação com a elevação da expectativa média de vida do idoso brasileiro. Segundo dados da Organização Mundial da Saúde (OMS), até 2025 o Brasil será o sexto país do mundo em número de idosos 2. Considerada uma população crescentemente vulnerável a riscos de doenças crônicas e a situações críticas durante suas atividades diárias, as pessoas idosas enfrentam a possibilidade de desenvolver sequelas, iniquidades e fragilidades. Além disso, destaca-se o perfil de saúde dos idosos muito idosos como uma fonte valiosa de informações, possibilitando aos profissionais de saúde a promoção de políticas públicas, para aprimorem competências e diagnósticos clínicos, com intervenções abrangentes. Essas ações podem incluir o cuidado à saúde, o ensino clínico aos pacientes e o planejamento de práticas de gestão de cuidados em saúde, visando à promoção da saúde e qualidade de vida. Nessa perspectiva, incentivar a condução de pesquisas nacionais que deem visibilidade às necessidades de saúde da população idosa, com o objetivo de melhorar as condições de saúde e proporcionar um envelhecimento mais longo e saudável, com bem-estar e qualidade de vida. 3.

O conceito de envelhecimento sofreu diversas alterações ao longo dos anos, evoluindo de acordo com as crenças, atitudes, cultura, conhecimentos e relações sociais. Sabe-se que o envelhecer é considerado como um processo universal, complexo e contínuo ao longo da vida 4. Esse processo é caracterizado pelo declínio lento e insidioso das propriedades funcionais em nível celulares, teciduais e orgânicos, proporcionando a diminuição de algumas capacidades cognitivas, inclusive a rapidez de aprendizagem e memória. No entanto, o processo de envelhecimento é mais perceptível na velhice, já que é nessa fase que a deterioração progressiva do organismo tende a limitar o indivíduo idoso na realização de diversas atividades, tornando-o, na maioria das vezes, incapaz 5.

É salutar que os profissionais de saúde e gestores compreendam os diversos aspectos correlatos à pessoa idosa em seus contextos sociais, econômicos e culturais. Outrossim, destaca-se a necessidade de promover a saúde, enfatizando o estímulo ao protagonismo do autocuidado por parte dessas pessoas, bem como, a gestão de uma abordagem ampliada e compartilhada da clínica gestão de cuidados pelos profissionais de saúde dispensados à pessoa idosa 3.

Dentre as diversas atividades afetadas pelo envelhecimento, o trabalho é uma das mais significativas. O trabalho pode ser definido como uma atividade de caráter produtivo desenvolvida pelo homem, que requer esforço físico ou intelectual, remunerada ou não, que caracteriza uma ocupação profissional. Além disso, o trabalho para o ser humano tem importância fundamental, uma vez que demanda grande parte do tempo dos indivíduos, cuja dinâmica é uma questão social, que proporciona não somente ganhos materiais, como também espaço na construção da identidade e vínculos afetivos 1,5,6.

Na aposentadoria, o vínculo com o trabalho é interrompido de forma abrupta. Por um lado, a aposentadoria pode ser acompanhada de uma sensação de liberdade e euforia, encarada em seu contexto legal, como uma obrigação do Estado para auxiliar as pessoas que se encontram com as forças físicas enfraquecidas. Por outro lado, a ociosidade pode dar lugar a um sentimento de inutilidade, improdutividade, solidão, dentre outros, desencadeando um desequilíbrio emocional e social 5,6. Nessa etapa da vida, outros fatores de risco podem ser somados ao idoso aposentado como sedentarismo, inadequados hábitos alimentares e dependência química. Nesse processo os comprometimentos emocionais e sociais, podem se manifestar em comportamentos e atitudes inesperadas e psicopatologias não antes apresentadas 6.

Uma das principais manifestações de cunho psicológico na população idosa é a depressão. Frequentemente o estresse é gatilho para desencadear a depressão em idosos, que se apresenta de forma complexa e é associada, sobretudo, a aspectos psicossociais, como solidão, aposentadoria, perda de emprego e aspectos não tratados durante a vida. Pode-se elencar o estresse como uma das consequências da aposentadoria, uma vez que a ruptura com o trabalho está associada à inutilidade, a incapacidade física e mental e a finitude da vida 6,7.

A depressão constitui-se de uma condição em que há variações conforme a fase da vida da pessoa que é por ela acometida. Dentre os sintomas que caracterizam a doença estão a diminuição do sono, perda de prazer nas atividades habituais, ruminações sobre o passado, perda de energia e de memória, isolamento social, humor depressivo, perda do interesse ou do prazer, agitação ou retardo psicomotor, fadiga, sentimentos de culpa, desesperança, diminuição de concentração, podendo estar associados a fatores biológicos, sociais e psicológicos, ainda sofrendo interferência de outras variáveis 4,6,8.

A depressão é considerada uma morbidade de interesse pelos profissionais de saúde e pelas autoridades de saúde pública, uma vez que seus efeitos deletérios são refletidos na saúde e na qualidade de vida dos idosos, realidade cada vez mais comum no contexto mundial, que pode impactar negativamente o sistema único de saúde e previdenciário, principalmente, com relação aos gastos onerosos em tratamento e manutenção de aposentadorias 9.

Assim, o papel dos profissionais de saúde é fundamental nesse processo de transição, principalmente os psicólogos, uma vez que estudam os sujeitos, contribuindo para o entendimento e elaboração dos sofrimentos que afetam os indivíduos. No período que antecede a aposentadoria, os sentimentos de insegurança, incapacidade, inutilidade e incertezas estão cercados de dúvidas nesta etapa de mudanças. Dessa forma, o psicólogo pode amenizar as angústias dos idosos, promovendo o bem estar e o desenvolvimento humano através da aplicação do seu conhecimento e técnicas específicas, preservando a autoestima e evitando o adoecimento 1.

Nesse contexto, o objetivo do estudo foi discuti a relação entre aposentadoria e depressão em idosos e promover uma reflexão teórica na perspectiva da clínica ampliada diante dessa transição, sendo oportuno para isso, a participação de uma equipe multiprofissional para enriquecer a discussão da problemática em questão.

## METODO

A presente pesquisa consiste numa reflexão teórica, tendo como critérios de inclusão, artigos científicos publicados entre os anos de 2012 a 2017, disponibilizados gratuitamente, com texto completo e em língua portuguesa, que discutem sobre aposentadoria e depressão, nos quais abordem discussões sobre as percepções e significados do trabalho na velhice, a transição para a aposentadoria, as relações entre aposentadoria e depressão do idoso e as contribuições da clínica ampliada na vida do idoso pré e pós-aposentadoria.

A busca dos artigos referenciais ocorreu na base de dados Scientific Electronic Library Online (SciELO) e Biblioteca Virtual da Saúde (BYS), por meio dos descritores em ciências da saúde (DeCS): idoso, trabalho, envelhecimento, aposentadoria e depressão. Foi utilizado o operador booleano AND para todas as buscas realizadas no SciELO através das associações: envelhecimento AND depressão; idoso AND depressão; idoso AND aposentadoria; envelhecimento AND aposentadoria; e depressão AND aposentadoria. Na BVS a busca foi realizada através do descritor "aposentadoria", associando com os assuntos principais: "idoso", "envelhecimento", "depressão" e "aposentadoria". Os critérios de exclusão, estudos que tratavam sobre aposentadoria por invalidez, que não apresentaram os critérios de inclusão ou que apareceram duplicados.

A seleção dos estudos referenciais, após a utilização dos descritores e filtros seguindo os critérios de inclusão, se deu em duas etapas: 1) leitura dos títulos e 2) leitura dos resumos. Após a leitura dos títulos, foram excluídos os estudos que não se enquadravam na temática. Os trabalhos pré-selecionados foram submetidos à leitura dos resumos, os quais foram excluídos aqueles que não se adequavam aos objetivos desta pesquisa. A coleta dos dados foi realizada em agosto de 2017.

## RESULTADOS

**Figura 1:**
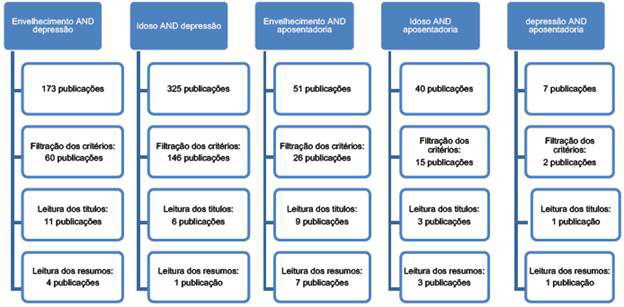
Fluxograma dos resultados da seleção dos estudos referenciais realizados na base de dados SciELO

Foram localizadas 6 599 publicações com os descritores sugeridos neste estudo. Após a inserção dos critérios de inclusão restaram 375 publicações e por fim foram selecionados 35 artigos para a realização desta pesquisa, (Figuras 1 e 2).

**Figura 2:**
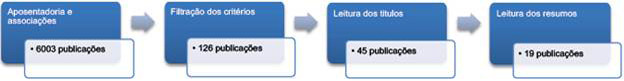
Fluxograma dos resultados da seleção dos estudos referenciais realizados na base de dados BVS Em relação ao ano de publicação, a maior quantidade de artigos foi publicada em 2014 e 2015 com 8 artigos em cada ano, seguido pelo ano de 2016, com 7 artigos publicados.

Sobre a metodologia empregada nos artigos referenciais, temos: 25 originais, 9 revisões e 1 estudo de caso. Nessa pesquisa, houve destaque para a Revista Brasileira de Geriatria e Gerontologia com 3 publicações, seguido pelos periódicos Psicologia & Sociedade, Psicologia: Ciência e profissão, Revista Kairós Gerontologia e Revista Psicologia: Organizações e Trabalho com 2 publicações cada. Sobre os autores presentes neste estudo, podemos destacar Lúcia Helena de Freitas Pinho França, Cristineide Leandro-França, Gustavo Silva Menezes, Sheila Giardini Murta e Maria Do Carmo Lourenço Haddad.

## DISCUSSÃO

Os sentimentos e percepções sobre velhice e trabalho são essenciais para predizer como o indivíduo enfrentará a ruptura com as atividades laborais e como se comportará diante das perdas e ganhos da aposentadoria.

Pesquisas realizadas com funcionários de universidades reafirmam que existem diversos fatores que podem contribuir para a concepção dos sentimentos frente à aposentadoria. Para os participantes, as vantagens na aposentadoria, podem ser o tempo livre para prática de exercícios e atividades culturais, viagens e maior dedicação à família e ao lazer. No entanto, alguns indivíduos relatam a ruptura do vínculo com colegas trabalho e a perda salarial e benefícios como principais desvantagens 10-12.

Para os autores, o contexto social, ocupacional e econômico contribui para a passagem do aposentado por esse processo. Segundo o estudo, os indivíduos casados deram maior significado ao tempo dedicado ao relacionamento e a família após aposentadoria. Aqueles que possuíam maior escolaridade perceberam a aposentadoria como um novo recomeço, enquanto os que tinham maiores salários conferiram menos importância às atividades de lazer e novos investimentos, visto que essa realidade está presente em seu cotidiano 12. De fato, vários significados podem ser percebidos por idosos aposentados. Os sentimentos de continuidade foram relatados por idosos que continuaram a exercer atividades laborais mesmo após a aposentadoria, a readaptação à nova fase da vida, traduzidos por novos planos e atividades e adaptações ao contexto familiar, e os sentimentos ambivalentes de perdas e ganhos no período pós-aposentadoria 13.

Resultados semelhantes foram encontrados por mulheres 14, elas afirmam que mesmo não havendo consenso na literatura sobre a adaptação na aposentadoria por homens e mulheres, ambos os sexos defendem que um período pós-carreira de sucesso está relacionado a ter uma boa saúde, ao planejamento no período pré-aposentadoria, a boa renda e as motivações com atividades prazerosas e lazer. Alguns fatores contribuem para uma aposentadoria satisfatória, tais como melhor saúde física e psicológica, prática regular de atividade física, consumo moderado de álcool, extinção do hábito de fumar, aumento do nível de escolaridade e ser casado.

Muitos aposentados revelam o desejam de voltar a atenção para a família após o desligamento com o trabalho. No entanto, um estudo realizado em Portugal, demostra que o ajustamento familiar na aposentadoria pode adquirir aspectos negativos, especialmente se há histórico de problemas de relacionamento na história conjugal. Culturalmente, na população portuguesa, o casamento é feito com parceiros mais velhos, sendo assim o homem aposenta-se primeiro. Esse cenário pode promover um sentimento de intrusão no espaço anteriormente administrado por mulheres. Nesse sentido, pode haver uma readaptação difícil expressada através de comportamentos hostis, isolamento, ansiedade e dificuldade em resolver os problemas. Assim, as autoras afirmam a necessidade de intervenção na família no momento da transição da aposentadoria e não somente no trabalhador 15.

Em um estudo 16, identificaram que a renda é um dos principais fatores que influencia o aposentado em continuar trabalhando. Os autores revelam que dispor de tempo para atividades prazerosas, mas não ter uma renda suficiente para realizar tais atividades gera o sentimento de frustração. A necessidade de reentrada no mercado de trabalho, exclusivamente, como forma de complemento da renda familiar é um dos grandes temores dos idosos aposentados. Dessa forma, se a aposentadoria estiver associada ao baixo poder aquisitivo, essa fase torna-se um grande momento de tensão, contribuindo também para a baixa autoestima e para o medo e frustração em não dispor de remuneração suficiente para as necessidades básicas, para ajudar a família e para concretizar os sonhos e planos.

Situação semelhante é descrita em pesquisa 17 com com trabalhadores informais da construção civil. A autora revela que todos os entrevistados continuaram a exercer atividades laborais após a aposentadoria devido à necessidade de complemento da renda. Essa realidade é vivenciada em maioria pelos homens, pois assumem a exigência social de provedor da família. Tal necessidade é agravada pela dificuldade de inserção no mercado de trabalho pelos jovens, pela gravidez de filhas adolescentes, por doença de parentes próximos ou pela inclusão de agregados no núcleo familiar.

Apesar da reinserção obrigatória no trabalho provocar sentimentos negativos no aposentado, 18 o trabalho contribui positivamente no combate à depressão, incapacidade e fragilidade em aposentados. Assim, idosos que trabalham mantem o bem-estar, bom nível de cognição, além de independência e autonomia na realização das atividades diárias. No entanto, a opção de continuar trabalhando requer cuidado e adequações para minimizar e evitar os danos indesejáveis decorrentes da função. Para isso, faz-se necessário investimento nessa parcela da população, especialmente, e, em relação à função musculoesquelética e cardiorrespiratória, além de políticas de promoção de saúde e de incentivo à prática de atividades físicas no ambiente de trabalho.

Estudo 12 demostra que o tempo de serviço pode contribuir negativamente para o processo de aposentadoria, visto que o trabalho e a instituição ocupam grande espaço na vida dos indivíduos, principalmente pela quebra dos vínculos pessoais construídos ao longo dos anos. Assim, à medida que o envelhecimento progride, pode haver uma dificuldade dos idosos em aceitar a aposentadoria como um processo natural. Nessa perspectiva, a importância de programas de preparo para a aposentadoria e a atuação do psicólogo faz-se primordial para que o indivíduo perceba essa nova identidade e busque outras fontes de prazer além do trabalho 12.

Estudo realizado com 1656 idosos revela que a participação em grupos de convivência mostrou-se como fator protetor para a depressão, uma vez que a rede de apoio social é um importante instrumento na manutenção da saúde e no combate aos sintomas depressivos 8. No entanto, uma pesquisa realizada com servidores de uma universidade revela que a adesão em programas de preparo para a aposentadoria é pequena, uma vez que a maioria dos entrevistados (48,8%) afirma o desejo de continuar trabalhando após a aposentadoria. Para as autoras, a rejeição da aposentadoria está ligada ao fato do trabalho não ser apenas uma fonte de renda, mas uma identidade social, em que o indivíduo organiza sua rotina, estabelece planos, exerce sua criatividade e constrói laços afetivos 19.

Em um estudo 7, comparando o desempenho de papéis ocupacionais em idosos com e sem sintomas depressivos, considerou-se que idosos que não têm sintomas de depressão apresentam a continuidade no serviço doméstico e em atividades voluntárias, bem como o planejamento para atividades de lazer. Segundo os autores, os dois grupos apresentavam o desejo de retomar algumas atividades perdidas, desmistificando o conceito de idoso como um indivíduo sem desejo e vontade. No entanto, a continuidade de projetos depende tanto da vontade do indivíduo quanto da rede de apoio e do contexto social o qual está inserido.

Uma intervenção breve acompanhada de follow-up por 11 meses com trabalhadoras em fase de pré-aposentadoria 20, revela que houve mudanças nos comportamentos das participantes em relação a rede de apoio social, planejamento de finanças e, principalmente, nos cuidados com a saúde. Assim, tornaram-se rotineiras as práticas de atividade física, cuidados com a alimentação e realização de exames médicos. A preocupação com a saúde é importante para uma aposentadoria bem-sucedida, uma vez que é possível reduzir o risco de doenças crônicas que prejudicam a qualidade vida na velhice dessas mulheres.

Muitos idosos acabam se sentido excluído pela sociedade, e acabam protelando sua aposentadoria, e optam pela permanência no mercado de trabalho 21. Assim, tornam-se cada vez mais necessários programas de intervenção e preparação para aposentadoria, uma vez que essas medidas são de fácil aplicação e custo econômico baixo. Dessa forma, além de contribuir para a promoção de saúde dos trabalhadores nas organizações, essas ações atendem as demandas previstas na Politica Nacional do Idoso 20. Ademais, destaca-se a importância do psicólogo como facilitador desse processo, por meio da orientação profissional, na qual a construção de novas escolhas e projetos, redimensionando a percepção sobre o envelhecimento e o trabalho, seja capaz de promover qualidade de vida na busca de uma aposentadoria bem sucedida 22.

O aumento da idade média da população demanda que os profissionais de saúde estejam capacitados para oferecer cuidados clínicos ampliados, alinhados com as políticas de saúde pública e o panorama epidemiológico. Além disso, é necessário adotar práticas dialógicas, visando a atender a um número crescente de indivíduos. Esse processo implica na avaliação dos serviços de saúde, na consideração da satisfação dos usuários, na implementação de intervenções, assim como no acompanhamento e rastreamento das condições de saúde da população, e na vigilância à saúde 23.

A clínica ampliada emerge como uma das orientações preconizadas pela Política Nacional de Humanização, visando aprimorar a abordagem na prestação de serviços de saúde. Ampliar a clínica significa potencializar a autonomia não apenas do usuário do sistema de saúde, mas também de suas famílias e da comunidade em geral. Este conceito implica na integração de profissionais de saúde de diversas áreas, colaborando conjuntamente na busca por cuidados e tratamentos personalizados, estabelecendo vínculos sólidos com os usuários. Nesse contexto, a vulnerabilidade e os riscos individuais são criteriosamente considerados, e o processo diagnóstico não se limita ao conhecimento especializado dos profissionais clínicos, mas incorpora também a história e singularidade de cada pessoa sob cuidado 24.

Nessa perspectiva, a clínica ampliada contribui para uma abordagem mais integral das pessoas, uma vez que não se restringe apenas ao manejo clínico dos sinais e sintomas. Ela também considera suas condições de vida e saúde, seus riscos, vulnerabilidades e necessidades de saúde que podem causar impactos negativos e originar doenças negligenciadas. Essas condições não só comprometem as relações interpessoais, mas também enfraquecem a autonomia dos sujeitos envolvidos nesse processo de saúde-doença.

É licito salientar o papel da clínica ampliada no desenvolvimento de uma abordagem holística e integral, tendo em vista a transição dos idosos para aposentadoria e como implicações deletérias estão associadas a esta fase da vida, sendo muitas vezes acarretada de desordens clínicas, como a depressão, tão comum nesse período de transição e pós-transição. Na perspectiva da velhice, isso implica em abordar não apenas os aspectos físicos, mas também os psicossociais, emocionais, culturais dos idosos, familiares, bem como, de apoio social, pensando-se em alcançar um equilíbrio da saúde e melhorar a qualidade de vida e bem-estar dos idosos aposentados.

A clínica ampliada enquanto uma abordagem de inovação tecnológica na perspectiva da saúde, visa a extrapolar a abordagem tradicional, indo além dos aspectos biologicistas. Além de incluir em sua cerne aspectos psicossociais e culturais, ambientais, familiares e da sociedade dos quais estas pessoas estão inseridas. Sendo entendida como uma visão mais compreensiva e amplificada das suas condições de vida e saúde por trás de uma roupagem, que envolve multifacetados campos e contextos para se alcançar diagnósticos consideráveis para a saúde das pessoas.

Perspectivas diversas a respeito da clínica tradicional, incluem abordagens puramente sintomatológicas e patológicas (envolve apenas as doenças). No entanto, a clínica ampliada coloca o paciente como protagonista ativa de seu próprio processo de cuidado ou tratamento. Isso implica em envolvê-los nas decisões clínicas acerca do seu próprio cuidado (autocuidado). Na qual os pacientes colocam em destaque suas vivências e conhecimentos a respeito de sua própria saúde. Nesse sentido, a aplicação da abordagem da clínica ampliada pelos profissionais de saúde tem como ênfase escuta ativa, sensível e compartilhada com os pacientes e constitui uma relação mutua de confiança entre o profissional e o paciente, na qual evidencia-se o compartilhamento de experiências vividas entre ambos os envolvidos visando-se estabelecer relações de parcerias para enfrentar os aspectos que influenciam a saúde do indivíduos, promovendo ,assim, a autonomia do paciente na gestão da clínica do seu cuidado.

A abordagem da clínica ampliada incluem práticas fundamentais para o cuidado integral do paciente. Evidencia-se a escuta qualificada e o acolhimento do paciente, elementos norteadores para compreensão das necessidades, sendo capaz de proporcionar um ambiente colaborativo e confiável. Além disso, a elaboração em equipe do plano clínico terapêutico representa marco crucial, uma vez que profissionais e pacientes compartilham estratégias para o tratamento, considerando não apenas aspectos clínicos, mas também os aspectos individuais, inerentes ao contexto do ser humano.

A clínica ampliada extrapola os limites tradicionais da saúde, promovendo ações intersetoriais que articulam áreas do conhecimento científico, tendo em vista a ampliação da clínica para prestação de cuidados mais abrangentes e eficazes. A intersetorialidade contribui para uma compreensão mais ampliada dos desafios e itinerários terapêuticos enfrentados pelo paciente. Nesse sentido, a educação em saúde é uma outra esfera da clínica ampliada, que visa capacitar o paciente para o autocuidado e promover a promoção, prevenção controle de agravos e doenças. Essa abordagem visa não apenas tratar doenças, mas visa também fortalecer a saúde e o bem-estar a longo do curso de vida.

Acompanhar e monitorar a evolução do pacientes ao longo do curso de vida, faz parte do contexto da clínica ampliada. Esses processos permitem realces no plano clínico terapêutico conforme as necessidades reais da pessoa, garantindo respostas adequadas às mudanças no estado de saúde do paciente. Por fim, e não menos importante a promoção da participação e autonomia do paciente é um princípio central da clínica ampliada. Incentivar participação ativa dos pacientes em decisões relacionadas à sua saúde é capaz de proporcionar maneiras de se assumir o papel ativo e contribui significativamente para resultados de cuidados mais incisivos e experiencias de saúde mais satisfatórias.

A abordagem da clínica ampliada visa a transmitir mais segurança aos pacientes, inclusive os idosos aposentados, uma vez que o envelhecimento é caracterizado como um processo multifacetado e individualizado para cada ser. Os profissionais de saúde devem estar preparados com o manejo clínico não apenas dos sinais e sintomas da doença, mas também todo o processo de saúde-doença-cuidado no qual esses idosos estão expostos nessa fase da vida, muitas vezes tão complexa, que envolve os determinantes sociais da saúde (aspectos socioeconômicos, renda, escolaridade, étnicos/raciais, culturais e comportamentais) , assim como os estilos de vida, condições de trabalho, acesso aos serviços de saúde, participação familiar e experiências de vida, os vínculos sociais, riscos à saúde, vulnerabilidades e necessidades de saúde, expectativas de vidas, bem como os anos potências de vida perdido do idosos, na perspectiva de que os profissionais de saúde envolvidos nesse processo, possam desenvolver uma atenção à saúde, bem mais humanística, crítica, reflexiva e ética, com discussão de casos clínicos entre os profissionais, e com a participação da pessoa doente, juntamente com seus familiares, e às vezes com participação da comunidade quando se faz necessário a participação de todos os atores envolvidos no processo de saúde-doença-cuidado para o fortalecimento de vínculos.

A transição para a aposentadoria é vista de forma individualizada e parte da subjetividade, do contexto social e da perspectiva de futuro de cada sujeito. A percepção negativa de envelhecimento, carregada de sentimentos ligados à inutilidade, incapacidade e a finitude da vida, associado com a maximização do significado de trabalho podem ser prejudiciais ao idoso em fase de aposentadoria.

O trabalho não constitui apenas uma fonte de renda, mas é nele que o sujeito cria laços afetivos, com colegas e seus familiares e com o público alvo do seu trabalho, organiza sua rotina, pessoal e ambiental, quase sempre, diária e projeta socialmente a sua identidade, sendo conhecido pelo seu trabalho, Fulano de tal órgão/setor. Normalmente, se passa mais tempo no trabalho do que na residência. Assim, a ruptura com o mercado de trabalho pode gerar isolamento social, dificuldades na readaptação familiar e falta de perspectiva na vida. Dessa forma, para muitos indivíduos essa transição pode vir carregada de sofrimento psíquico, resultado resultando em desordens emocionais e transtornos mentais.

A literatura aponta que após a saída do mercado de trabalho pode haver desarranjo na estrutura familiar, ociosidade, aumento de consultas médicas, dependência de álcool e outras drogas, que associados com os sentimentos de perda de identidade e inutilidade podem gerar depressão, diminuição na qualidade de vida e, em alguns casos, desencanto pela sua própria vida, sendo frequente as tentativas de suicídio.

No entanto, apesar da importância social e de saúde, ainda é pequeno o número de publicações na literatura brasileira que aborde a temática da depressão em idosos, especialmente nos aposentados. Também são escassas as pesquisas voltadas para a aposentadoria sob a ótica da clínica ampliada. A maioria dos estudos aborda temáticas que tratam das percepções e sentimentos do trabalhador em face da aposentadoria, mas negligenciam os aspectos da saúde mental.

A carência de material sobre a contribuição dos profissionais de saúde, na preparação no que diz respeito à aposentadoria, também revela uma limitação no campo de atuação desses profissionais. Geralmente, evidencia-se a atuação do psicólogo nas organizações que se limita as atividades voltadas para o setor de recursos humanos (de recrutamento, seleção e treinamento). Sendo assim, faz-se necessário a inserção do psicólogo nas decisões organizacionais das instituições públicas e privadas e nos Programas de Preparo para Aposentadoria. Assim como, o engajamento de outros profissionais da saúde na prestação de cuidados clínicos ampliados a essa população em questão.

Os PPAs devem produzir ações educativas, com o intuito de promover o debate sobre o envelhecimento como um processo natural e que trabalhe a percepção do sujeito em relação aos significados do trabalho e da aposentadoria. Dessa forma, será possível que o trabalhador passe por esse processo de forma tranquilo, a fim de garantir que no período pós-carreira ele possa descobrir suas potencialidades e explorar outras alternativas prazerosas.

Cabe ressaltar também, que estratégias de preparo para a transição do período pós-carreira fazem parte das exigências do Estatuto do Idoso e da Política Nacional do Idoso. Dessa forma, a literatura aponta a necessidade do preparo continuado ao trabalhador e não somente às vésperas da aposentadoria. Portanto, estas ações de intervenção são essenciais para que o sujeito idoso possa desfrutar de uma aposentadoria bem-sucedida e sem grandes impactos na sua saúde.

No entanto, apesar da importância social e de saúde, ainda é pequeno o número de publicações na literatura brasileira abordando a temática da depressão em idosos, especialmente nos aposentados. Também, são escassas as pesquisas voltadas para a aposentadoria sob a ótica da clínica ampliada. A maioria dos estudos aborda temáticas que tratam das percepções e sentimentos do trabalhador em face da aposentadoria, mas negligenciam os aspectos da saúde mental.

Por outra perspectiva, o multiprofissionalismo quando desenvolvido através de ações colaborativas entre diferentes profissionais de saúde, pode contribuir com cuidados mais amplos e com competências clínicas mais assertivas para essa fase da vida, tão complexa. Muitas vezes requer o engajamento de toda a equipe interdisciplinar, desde de médicos, enfermeiros, fisioterapeutas, psicólogos, assistentes sociais, educadores físicos, entre outros. A integração desses profissionais corrobora com uma assistência à saúde mais completa, humanizada e que atenda as reais necessidades do idoso, além de possibilitar intervenções mais adequadas e centradas no paciente. Os profissionais de saúde podem também, dá suporte emocional, através do ensino clínico, com orientações acerca de estilos de vida saudáveis e engajar na colaboração de novos signos, significados e auxiliar no incremento de atividades na fase da aposentadoria.

O processo de aposentadoria na vida dos idosos é uma reflexão vital no contexto do envelhecimento humano contemporâneo. A aposentadoria dos idosos não se limita na descontinuidade de suas atividades laborativas no mercado de trabalho, mas implica em criar estratégias e ambientes propícios de trabalhos alternativos, que promovam seu bem-estar físico e mental, e que verdadeiramente promovam a participação efetiva dos idosos na sociedade, para que não sejam esquecidos como fonte motriz geradora de trabalho e protagonistas da economia de determinado país.

A tríade idosos, aposentadoria e o mundo do trabalho é extremamente complexa, não apenas por causa da importância para economia, mas por englobar repercussões significativas no mercado de trabalho. À medida que a população envelhece, impactos significativos na previdência social e economia em geral são constantemente acentuados, tornando a aposentadoria um fardo para toda a sociedade. Superar os desafios dos estigmas econômicos causadas pela aposentadoria precoce, muitas vezes, é o caminho a ser seguido pelo países desenvolvidos, isso pode refletir nos países em desenvolvimento na prestação cuidados clínicos de saúde ampliados. Ao repensar pressões econômicas causadas pela aposentadoria, requer o engajamento de formuladores de políticas públicas no subsídio de ambientes de trabalho inclusivos diante do crescimento da população idosa, afim de prolongar a vida profissional, na perspectiva de trazer dignos benefícios previdenciários, e possa garantir a permanência dos idosos no mundo do trabalho, apresentando reflexos significativos ainda na economia. Isso pode contribuir com o envolvimento de idosos altamente habilitados e com acúmulos de experiências notáveis, como parte do envelhecimento bem sucedido.

O profissional de saúde que trabalha em equipe na assistência as pessoas, deve reconsiderar, formular, e aplicar práticas clínicas no seu cotidiano diário, muitas vezes, esses cuidados estejam embasados em evidências científicas, a fim de subsidiar políticas públicas de saúde para uma população que está em constante crescimento e necessitam de assistência clínica especializada. Dentre as justificativas, entendemos a importância de revisar e aprimorar as práticas clínicas na assistência a pessoa idosa, pois a procura pelo embasamento em evidências científicas favorece mais cuidados clínicos especializados e eficazes, além disso, promove uma melhor previsão de morbidade relacionada aos desafios de saúde mais comuns.

Aprimorar o cuidado a partir da implementação de protocolos de cuidados clínicos embasados na clínica ampliada e compartilhada com os profissionais de saúde, numa perspectiva interprofissional multiprofissional, torna-a mais um diferencial para as vantagens dos pacientes. Portanto, os protocolos, principalmente como os manuais e as cartilhas clínicas: promovem, previnem, reabilitam as pessoas e, também, atendem ao atendimento humanístico, clínico e baseado em evidências científicas. As diretrizes clínicas facilitam o trabalho do profissional de saúde, a quem é confiado permitir contribuir para o bem-estar e a qualidade de vida das pessoas. E respaldam-se embasados pelo PNH -Programa Nacional de Humanização na Assistência à Saúde -, do Humaniza S US, do Ministério da Saúde.

A partir da perspectiva da Clínica Ampliada, é possível mitigar os efeitos negativos da aposentadoria em idosos por meio de uma abordagem holística e interdisciplinar. Isso envolve a integração de diferentes profissionais de saúde, como psicólogos, assistentes sociais, enfermeiros, educadores físicos, fisioterapeutas, terapeutas ocupacionais e médicos, para oferecer um suporte abrangente aos idosos durante essa transição. O foco deve ser na promoção da saúde mental e emocional, na manutenção do bem-estar físico e na reinserção social.

Um aspecto crucial é o apoio psicológico, que pode ajudar os idosos a lidar com os sentimentos de perda de identidade e inutilidade, além de proporcionar estratégias para enfrentar o isolamento social e a depressão. Os assistentes sociais podem auxiliar na identificação de recursos comunitários e na construção de redes de apoio social, ajudando os idosos a se envolverem em atividades significativas e a se sentirem parte de uma comunidade.

Além disso, os profissionais de saúde podem oferecer orientação sobre estilo de vida saudável, incentivar a prática de atividades físicas e promover hábitos alimentares adequados para garantir a saúde física e mental dos idosos. A terapia ocupacional também desempenha um papel importante, ajudando os idosos a encontrar novas formas de ocupação e propósito após a aposentadoria, seja por meio de hobbies, voluntariado ou educação continuada.

Por fim, é essencial que os serviços de saúde adotem uma abordagem centrada no paciente, valorizando suas necessidades, desejos e experiências individuais. Isso inclui a criação de espaços de escuta ativa, sensível e empática, onde os idosos se sintam ouvidos e respeitados em suas preocupações e aspirações. Ao adotar essa abordagem ampliada e integrada, é possível promover um envelhecimento saudável e bem sucedido, mesmo após a aposentadoria ♣
